# Nanoenergetic Materials: From Materials to Applications

**DOI:** 10.3390/nano14191574

**Published:** 2024-09-29

**Authors:** Rajagopalan Thiruvengadathan, Anqi Wang

**Affiliations:** 1 Department of Engineering and Technology, Southern Utah University, Cedar City, UT 84720, USA; 2 Department of Mechanical and Mechatronics Engineering, University of Waterloo, 200 University Avenue West, Waterloo, ON N2L 3G1, Canada; anqi.wang2@uwaterloo.ca

**Keywords:** nanothermites, combustion, graphene, nanoenergetic materials, propellants

## Abstract

Both nanoscience and nanotechnology have undoubtedly contributed significantly to the development of thermite-based nanoenergetic materials (NEMs) with tunable and tailorable combustion performance and their subsequent integration into devices. Specifically, this review article reflects the immense paybacks in designing and fabricating ordered/disordered assembly of energetic materials over multiple length scales (from nano- to milli-scales) in terms of realization of desired reaction rates and sensitivity. Besides presenting a critical review of present advancements made in the synthesis of NEMs, this article touches upon aspects related to various applications concomitantly. The article concludes with the author’s summary of the insurmountable challenges and the road ahead toward the deployment of nanoenergetic materials in practical applications. The real challenge lies in the ability to preserve the self-assembly of fuel and oxidizer nanoparticles achieved at the nanoscale while synthesizing macroscale energetic formulations using advanced fabrication techniques both in bulk and thin film forms. Most importantly, these self-assembled NEMs have to exhibit excellent combustion performance at reduced sensitivity to external stimuli such as electrostatic discharge (ESD), friction and impact.

## 1. Introduction

Energetic materials (EMs) release immense quantities of energy upon violent chemical reactions between oxidizer and fuel. These materials can be grouped into two categories. One is called monomolecular EMs, composed of fuel and oxidizer constituents within the same molecule. Some examples of this class of materials are the perchlorates, nitro-explosives, nitrate- and nitroamine-based explosives [1]. The second broad category is the composite EMs. Here, the oxidizer and the fuel components are mixed together to form the composite. Some prominent examples of this category are Al/Bi_2_O_3_, Al/MoO_3_, Al/CuO, and Al/Fe_2_O_3_, wherein the aluminum metal is the fuel, and the metal oxide is the oxidizer [2,3,4,5,6]. Yet another prominent example of composite energetic material is the solid propellant which is typically composed of monomolecular EMs, polymeric binders and catalysts [7,8,9,10]. 

The two main metrics that define the efficiency of EMs are energy density and the energy release rate. Monomolecular EMs exhibit high reaction rates as opposed to energetic composites. The rate of reaction in monomolecular EMs is primarily the kinetics of dissociation and evolution of chemical bonds (chemical kinetics). In contrast, the reaction rate in energetic composites is influenced by mass transport (diffusion kinetics) and hence it is lower in comparison to monomolecular EMs. However, the energy densities of monomolecular EMs are lower in comparison to composite EMs. It is often desired to produce EMs that exhibit high energy density and high energy release rate. 

Research works reported in the past two decades have amply established the potential of nanoscience and nanotechnology beyond any doubt to realize the production of EMs that can exhibit high energy densities and high reaction rates [4,11,12,13,14,15]. The high energy density comes primarily from a suitable choice of chemical composition of oxidizer and fuel. The high rate of energy release is credited to both the nanoscale dimensions of oxidizer and fuel particles as well as the ability to ensure a high degree of interfacial contact between them through a suitable self-assembly approach, thereby drastically reducing the heat and the mass transfer length scales. A number of self-assembly approaches have been investigated to augment the interfacial contacts between the fuel and the oxidizer of nanoenergetic materials (NEMs) [3,6,16,17,18,19]. Scientific investigations on self-assembly have remained more or less qualitative in the sense that the published works report on the self-assembly mechanisms and their correlation with combustion performance characteristics such as burn rate and pressurization rate. This class of composite EMs formed with either nanosized fuel or nanosized oxidizer or both oxidizer and the fuel in nanoscale dimensions is often called NEMs. Significantly, the ability to achieve tailorable yet desired combustion properties in such materials is the remarkable aspect that has provided the wonderful opportunity for their deployment as primers, electric matches, microthrusters, and explosives, among others. However, there are a number of issues that need to be addressed to realize practical applications of NEMs in the real world. These include scalability, particle aggregation, stability and sensitivity to electrostatic discharge, impact and friction. 

The world of EMs is huge, and the wealth of the published literature is therefore humongous. This article is focused on summarizing the key developments over the past two decades. The review paper is conveniently written in four sections dealing with diverse topics within the broad domain of NEMs. Section 1 comprehensively discusses the surface passivation methods employed for Al nanoparticles and highlights the key achievements. Section 2 presents a brief note on the reaction mechanisms. Section 3 deals with the present trends in self-assembled nanothermites without the inclusion of graphene and its derivatives. Section 4 summarizes the recent developments in graphene-based self-assembled nanothermite formulations. Section 5 reviews the recent developments in the application aspects of NEMs. The application of nanostructured additives including graphene and graphene-based materials as catalysts to enhance the combustion performance of propellants is discussed in this section; it also highlights the efforts on microdevices and additive manufacturing methods for NEMs. Specifically, this article focusses on the effects of the addition of graphene and its derivatives on ignition, combustion performance, thermal stability and sensitivity. The concluding section summarizes the challenges that persist from the viewpoint of fundamental science and applications. This section also highlights the opportunities for future research in the world of NEMs. 

## 2. Aluminum Nanoparticles and Surface Passivation

Conventional monomolecular EMs are more often mixed with metal particles to enhance the total energy density of the resulting composites. The heat of the reaction of metals in pure oxygen per unit volume is 5 to 12 times larger than that of monomolecular EMs such as CL-20, RDX and TNT. Among the metallic fuels, aluminum is more commonly used though the highest value of volumetric energy density of 138 kJ/cm^3^ is exhibited by boron. This is because of the following facts. Firstly, the native oxide layer (B_2_O_3_) present as the shell inhibits and delays the combustion reaction of the metal boron core with air. Secondly, the ignition temperature of boron particles is 1500–1950 K independent of particle size, while the melting point of the oxidation product B_2_O_3_ is only 723 K, which becomes liquid and hinders the combustion reaction. Thirdly, the combustion of boron particles in hydrogen-containing ambience leads to the formation of metastable HBO_2_ species, consequently lowering the energy release [20,21,22,23]. On the other hand, Al particles are commonly used in a number of applications including propulsion, explosions and pyrotechnics. However, Al nanoparticles are more attractive compared to their micron-sized Al particles. The two main fundamental issues associated with Al micron particles are higher ignition temperatures of up to 2077 °C for 100 μm and their inherent tendency to agglomerate prior to ignition [24]. On the other hand, the melting point of Al nanoparticles is size-dependent (decreases from 727 °C for 100 nm to 400 °C at 3 nm). Al nanoparticles are highly reactive compared to micron-sized Al particles, attributed to the greater number of surface atoms, higher surface area and the greater surface energy of these atoms. Thus, these nanoparticles exhibit lower ignition temperatures in comparison to their micron-sized counterparts [1]. Interested readers on the synthesis methods of metal nanoparticles can refer to the well-written article by Sundaram et al. [1]. 

However, the fundamental issue that hinders the employment of Al nanoparticles in applications is the occurrence of sintering and consequent aggregation due to an inherently slow diffusion process. Specifically, this problem can result in erratic combustion and/or sometimes motor failure in case of propellant application. Secondly, due to the presence of a passivating native oxide layer of 2–4 nm, the active metallic Al content reduces significantly with decreasing particle size [25]. For example, Al nanoparticles with an average particle size (APS) of 80 nm have an active metal content of about 70 to 80 wt.% depending on the thickness of the Al_2_O_3_ shell (2 to 4 nm). On the other hand, Al nanoparticles with APS of 50 nm have a metallic content of about 40 to 50 wt.%. Thus, the fraction of dead mass due to the presence of an Al_2_O_3_ shell increases significantly with decreasing nanoparticle size, thereby reducing the overall heat of the reaction. Apart from the above-mentioned issues, nascent Al nanoparticles are ignited spontaneously when exposed to air at room temperature. On the contrary, the presence of an ultra-thin native oxide layer enables to overcome serious safety issues during the handling and storage of these nanoparticles to a reasonable extent. So, it is evident that there is a tradeoff between the desire to realize higher metallic Al content (ensuring higher reactivity) and the desire to ensure safety during storage, handling and processing. However, the dead mass of Al_2_O_3_ does not contribute to the energy release during the combustion reaction. 

In recent years, substantial research efforts have been undertaken to replace the Al_2_O_3_ shell with energetic groups through suitable coating methods. Key experimental results are summarized here. Nickel-coated Al nanoparticles with APS of 237 nm were synthesized with an active Al content of 53 wt.% only by electric explosion of Al-Ni wires [26,27]. The inherent process parameters of the production method did not permit tailoring the active Al content and the APS of nanoparticles and hence the combustion performance of Al was no better than Al nanoparticles passivated with an Al_2_O_3_ shell. In contrast, the synthesis of boron-coated Al nanoparticles revealed the active Al content was similar to that of Al_2_O_3_-passivated Al nanoparticles. Furthermore, the heat of reaction enhanced to 6232 kJ/kg from 5465 kJ/kg. Thus, boron coating is promising for application development. However, the issues related to scalability and the stability of boron-coated Al nanoparticles need to be addressed. 

Al nanoparticles passivated with carbon terminated surface with APS of 80 nm and a shell thickness of 1–3 nm were produced using laser ablation and arc-discharge techniques under argon/ethylene ambience [28,29,30]. Carbon-coated Al nanoparticles were thermally stable up to 700 °C and oxidized around 800 °C, which is approximately the oxidation temperature of carbon. Other techniques employed to produce a narrow size distribution of carbon-coated Al nanoparticles in the size range of 20 to 50 nm were microarc discharge and laser heating. Interestingly, these nanoparticles produced by laser heating exhibited a lower onset oxidation temperature of 495 °C and an exothermic peak was observed at 556 °C, which is lower compared to neat aluminum nanoparticles.

Coating of Al nanoparticles with organic materials is an attractive scheme for a number of reasons. Organic materials are combustible in oxidizing ambience and hence, it is possible to enhance the total heat of the reaction. Organic materials decompose at lower temperatures, thereby facilitating the higher reaction rate of Al nanoparticles. Solid propellants have organic materials in the form of plasticizers and binders. Hence, organic coating as a surface passivation layer of Al nanoparticles can provide better chemical compatibility with the constituents of propellants and overcome the processing difficulties normally associated with inorganic-coated Al nanoparticles. 

Passivation of Al nanoparticles with fluorine-based chemicals such as perfluoroalkyl carboxylic acids has been attempted to exploit the unique advantage of the high reactivity of fluorine species (high enthalpy of Al–F reaction) [31,32]. Specifically, the adiabatic flame temperature of Al-fluorine reaction is 4400 K, which is higher than that of Al-oxygen reaction (4000 K). The as-synthesized Al nanoparticles with an average core size of 5 nm contained an active Al content of 15.4 wt.%, which is extremely low. Fluorine is highly reactive and therefore, it is possible that fluorine atoms may react and etch the Al_2_O_3_ shell at a much lower temperature well before the melting of the Al core. This would enable better accessibility of Al core to external oxidizing species for propelling a complete solid-state reaction, and also possibly enhance the reaction rate. In fact, such an observation on the reaction between fluorine species in fluoropolymers (tetrafluoroethylene, hexafluoropropylene, and vinylidene fluoride (THV) and PTFE) and Al_2_O_3_ shell has been reported in the literature [10,33,34,35,36]. Instead of removing the Al_2_O_3_ layer, it would be very beneficial to coat it with fluoropolymers. Most importantly, the inherent hydrophobicity of fluoropolymers would also be a boon for improving the stability of Al nanoparticles against humidity. 

Experimental efforts to passivate Al nanoparticles with organic chemical agents such as oleic and stearic acids have also been reported in the literature [27,37,38]. With oleic acid coating, the weight percent of active Al content is reported to be only 40% while that of organic coating is 35 wt.%. The remaining 25 wt.% is that of Al_2_O_3_ formed due to the presence of oxygen atoms in the oleic acid molecule. A similar observation with reduced active Al content is seen with stearic acid. Among different organic materials used for in situ passivation of chemically synthesized Al nanoparticles with an APS of 30 nm, alkyl-substituted epoxides appear to be most promising on the basis of 96 wt.% active Al content estimated from chemical analysis of these nanoparticles post-synthesis [39,40]. This epoxide coating not only caps the surface but also stabilizes against further oxidation. Without this alkyl-substituted epoxide coating, the Al nanoparticles were pyrophoric in nature. 

**Figure 1:**
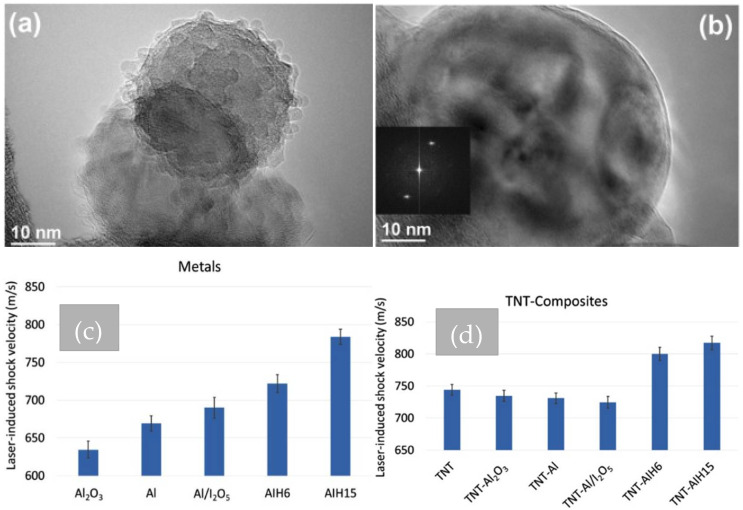
(**a**,**b**) HRTEM images of Al-AIH core–shell nanoparticles. (**c**,**d**) Laser-induced shock velocities for the metal- and TNT-based composites. Reprinted with permission from Jennifer L. Gottfried et al., *Sci Rep* 8, (2018) 8036 [41]. Copyright © 2018, Springer Nature.

Smith et al. reported an approach wherein the Al_2_O_3_ shell was replaced with an aluminum iodate hexahydrate (AIH) layer via the addition of Al particles to concentric iodic acid [41,42,43]. These Al (core)/AIH (shell) particles were found to be very reactive and exhibited a combustion wave speed of 3200 m/s. Subsequently, an interesting study was carried out to understand AIH’s contribution to reaction pathways (detonation as opposed to deflagration) using the laser-induced air shock from EMs (LASEM) technique [41]. An energetic composite of trinitrotoluene (TNT) mixed with Al/AIH core–shell particles was prepared and tested in the lab. Figure 1a,b shows high-resolution transmission electron microscopic (TEM) images of Al/AIH core–shell nanoparticles recorded at different magnifications. The nodules projecting out in Figure 1a reflect the AIH shell. The spots seen in the diffraction pattern (recorded by taking the FFT) reveal the single crystalline nature of Al/AIH nanoparticles (see Figure 1b). The tests revealed high reactivity in both fast (detonation) and slow (deflagration) combustion modes, primarily due to achieving optimum fuel-to-oxygen ratio in this TNT–Al/AIH mixture. The average laser-induced shock velocities of pure metal additives and TNT samples with and without Al/AIH inclusions are shown in Figure 1c,d. 

Among various samples prepared in this work, composite samples with 15% AIH exhibit the highest shock wave velocity both with metal and TNT. It is significant to notice that the neat TNT has an oxygen balance of −74%. This work is commendable and unique in that it gives experimental evidence of the detonation of Al-based explosives without the presence of a diffusion-limited Al_2_O_3_ layer [41]. Given the promising potential of the AIH passivation layer, it is indeed necessary to understand the underlying mechanisms of thermal decomposition of AIH. Therefore, Rizzo et al. performed such a study to bridge the knowledge gap on different stages of thermal decomposition of pure AIH [44,45]. A detailed Raman spectroscopic study conducted in this work presents experimental evidence to define the decomposition process into three main stages initiated by a carbon dioxide (CO_2_) laser from 302 K to 606 K. The first stage at 375 K has been attributed to the loss of water (H_2_O) in the gaseous phase from the hexacoordinated Al ion. The second stage happened at 480 K with the release of diiodine pentoxide (I_2_O_5_) plus H_2_O gas. The final stage was observed at 580 K with the suggested release of iodine and oxygen species with the decomposition of diiodine pentoxide. Optical images were recorded continuously as a function of temperature. The enhanced release of oxygen in the third stage was seen from the ignition and combustion of AIH particles. Reduced ignition delay is indeed the potential impact of this fascinating fundamental study on the thermal decomposition of AIH behavior. Consequently, this is good for the development of advanced propellant-based ammunition systems. A similar approach to the removal of the Al_2_O_3_ shell with reactive iodine has been reported in I-rGO/Al/Bi_2_O_3_ composite to enhance the combustion characteristics and this has been discussed in the subsequent section on self-assembled graphene-based composites. 

The stability of the passivation layer at elevated temperatures is another important parameter that needs detailed investigations. For example, polymer-bonded explosives (PBX) have been processed in hot water (50–70 °C) as a medium in an effort to ensure safety during processing. It is obvious that one cannot utilize Al passivated with the Al_2_O_3_ layer having surface hydroxyl groups directly in the preparation of PBX. Furthermore, fluorine-based polymers are used as binders in aluminized PBX. It is important to ensure that there is good compatibility between the surface of Al nanoparticles and fluorine. The choice of polymer is therefore the key to energetic composites. Among several polymer candidates, the glycidyl azide polymer (GAP) is attractive owing to the presence of the active –N_3_ group as well as its ability to enhance stability against reaction with water. GAP is also expected to increase the mechanical integrity of PBX by facilitating superior adhesion. Keeping these objectives in mind, Zeng et al. synthesized Al/GAP core–shell nanoparticles via in situ grafting [46].

**Figure 2:**
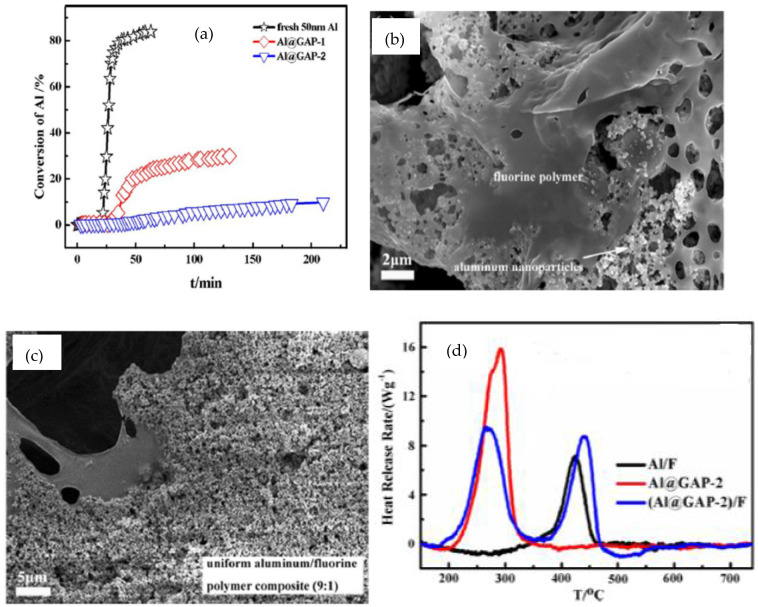
(**a**) Percent conversion of metallic Al estimated via conducting the aging test Al@GAP in water at 60 °C. SEM image of (**b**) neat Al–Fluorine polymeric composite showing non-uniform distribution of neat Al nanoparticles and (**c**) GAP-grafted Al–Fluorine composite nanoparticles showing homogeneous distribution of GAP-grafted Al nanoparticles and (**d**) heat of reaction obtained from DSC measurements. Reprinted with permission from Zeng et al., *J Mater Sci* 53, (2018) 12091 [46]. Copyright © 2018, Springer Nature.

Structural and compositional analysis with X-ray diffraction, scanning electron microscopy (SEM) Fourier transform infrared (FTIR) spectroscopy, Raman spectroscopy and X-ray photoelectron spectroscopy (XPS) methods confirmed the grafting of GAP onto the surface of Al nanoparticles using Toluene-2, 4-diisocyanate as evidenced by the formation of –O–(CO–NH–) chemical bonds. The contact angle of 142.4° measured for GAP-grafted Al nanoparticles further demonstrated the hydrophobic character. Both the neat and the Al/GAP nanoparticles were subjected to accelerated aging by stirring them in water at 60 °C continuously as a function of time. The percent conversion of Al upon reaction with water estimated by monitoring and measuring the hydrogen evolution is shown in Figure 2a. It is evident that GAP-grafted Al nanoparticles are more stable and exhibit resistance to oxidation upon reaction with water in comparison to neat Al nanoparticles. As seen in Figure 2a, samples with a thicker coating of GAP (sample: Al/GAP-2) showed better stability than those with a thinner GAP coating (sample: Al/GAP-1). These observations have been amply supported through the experimental observations from XPS measurements conducted before and after the aging test. The presence of –N_3_, –C=N– and –C=O groups in the spectrum for samples with GAP coating suggests the enhanced protection of Al nanoparticles from unwanted reactions with water. To evaluate the combustion performance of GAP-grafted Al nanoparticles and compare them with neat Al nanoparticles, the authors prepared fluorine-based composites by mixing them with fluorine-based polymer. SEM imaging clearly shows that the neat Al nanoparticles do not mix uniformly in fluoropolymer (Figure 2b). In contrast, SEM images confirm the homogeneous distribution of GAP-grafted Al nanoparticles fluoropolymer (Figure 2c). For faster and complete combustion, the miscibility of fuel in the oxidizer matrix and vice versa is extremely vital. In fact, complete and bright combustion with the shortest burn time was achieved for Al@GAP-2)/F composites as seen from the high-speed video recording. The total heat of reaction calculated from thermal analysis experiments using differential scanning calorimeter (DSC) (Figure 2d) is highest for composites prepared with GAP-grafted Al nanoparticles. In summary, the passivation of Al nanoparticles is the key to ensuring greater stability of Al nanoparticles, thereby ensuring better and more reliable combustion performance. Among the different coatings employed, halogen-coated Al nanoparticles are very attractive from the application perspective as well and this has been further discussed in the section on self-assembled graphene-based composites. However, the transition from the laboratory method to the industrial scale for passivation is yet to be established and demonstrated. 

## 3. A Brief Note on Reaction Mechanisms

Conventionally, nanothermite-based NEMs are produced in powder form, resulting in severe constraints for practical applications. Additionally, the high sensitivity of NEMs to ESD, friction and impact complicates the problem. Therefore, research efforts have been constantly undertaken to produce NEMs in the form of pellets [47], films [48], foams [49], membranes [50] and aerogels [19]. It is evident that the fuel and the oxidizer in all of these forms are packed into a higher density in comparison to that of powders. A well-known experimental observation is that the burn rate decreases with increasing percent theoretical maximum density (% TMD) [5,51]. These studies have concluded that convection dominates over conduction and radiation in heat transfer, leading to higher burn rates at low percent TMD as in powders. For example, this decreasing trend in burn rates has been reported for both self-assembled and unassembled nanothermites [5,51]. It is also seen that the pressurization rate does not show a monotonic trend with increasing TMD. Below 20% TMD, convection is the dominant mechanism and above 20%, conduction mechanism manifests and plays a significant role in comparison to conduction. At the same time, recent works have revealed that movement of condensed phase (i.e., solid or molten) material has also been observed and could play a role in effective heat transfer and propagating the reaction [52,53]. In fact, Egan et al. concluded that convection alone as a heat transfer mechanism cannot explain the combustion behavior at low percent TMD and established that movement of the condensed phase plays a significant role in the combustion propagation based on simple thermodynamic calculations [54].

Simultaneously, several computational modeling studies have been reported on the combustion reactions of nanothermites [47,55,56,57,58]. Phase changes, effects of ambient oxygen, decomposition of CuO and the presence of alumina shell were the key parameters considered in the study reported by Baijot et al. [55]. However, the crucial issue of reaction front propagation was not addressed in their work. Multiphysics-based models employing heat equations were subsequently developed to study the combustion propagation of compressed pellets. Knapp et al. laid out a novel approach to develop a hotspot model wherein the authors took into account the effects of equivalence ratio (ϕ) and particle sizes of oxidizer and fuel [58]. However, to address the role of convection in combustion propagation reactions, modeling efforts must consider inherent porosity ubiquitously present in nanothermite pellets. Epps et al. considered such an approach to report a continuum scale model to study the reaction propagation in the Al/CuO pellet [56]. This model is essentially a two-phase (gas and non-gas (condensed and solid phase)) numerical model based on Darcy’s law and the transient heat equation. At the same time, the fundamental physics involving mass, momentum and heat transfer were considered. Though the model agrees with the general experimental observations of both conduction and convection regimes as a function of packing density, the burning behavior at low percent TMD is not quantitatively captured by the model, as the authors reiterate. Since the model is reported to be sensitive to the pore structure (shape and size distribution), more accurate quantitative details of pore structure within a compressed pellet must be incorporated into the numerical model. Concluding this section, one can state that there is more scope for research related to theoretical and computational models of combustion reactions. Methodologies based on data science and artificial intelligence may be employed to unearth the mechanism of combustion reactions, considering the available experimental data reported in the literature for nanothermite formulations. 

## 4. Arrested Reactive Milling for NEMs

Arrested Reaction Milling (ARM) is a dedicated and sophisticated procedure that has been employed to synthesize NEMs with tunable combustion characteristics [59,60]. This method combines features of reactive milling and nanotechnology to create NEMs with controlled reactivity. The milling process is carried out in a controlled environment to prevent unwanted reactions or contamination [61]. During the milling progression, the precursors are exposed to mechanical forces that induce reactions. The objective is to commence the reaction between the components while simultaneously controlling the conditions to manage the reaction progress. Precise control of the milling parameters such as time, temperature, and environment to halt the reaction at a specific point is the key to proper synthesis of NEMs. The word “arrested” in the milling process signifies this part [62]. Of course, a lack of control during the synthesis would obviously give rise to undesirable and intense reactions. The capability to impede the reaction at exact times allows for the fabrication of NEMs with tailored properties for specific applications, such as high-energy-density explosives or propellants.

ARM has been proven to fabricate various NEMs, such as Al/MoO_3_, Al/Fe_2_O_3_, Al/CuO, etc. [63,64] in the past decades. In recent years, the research has shifted towards more specific applications and properties. Nguyen et al. [65] added a second milling process that utilized softer conditions such as reduced rotation rate, smaller beads, and additional liquid as agents, resulting in a more homogeneous particle size distribution, and better flowability without hindering the reactivity of the material. Mursalat et al. [66] prepared a porous Al/CuO nanocomposite using acetonitrile as a milling agent, giving a distinct low-temperature exothermic peak around 600 K. It was found that a redox reaction pathway of released gaseous oxygen from CuO decomposition triggered the low-temperature exothermic peak as the free molecules transported to the surface of surrounding Al particles. This gas–solid reaction hindered the formation of intermetallic Al-Cu, leading to the formation of nanometer-scale metallic copper, which accelerated the ignition and led to higher propagation velocity.

ARM is also more utilized for NEM systems with complex compositions due to its advantages of scalability, low cost, and strong mechanical effect that can simply blend materials that cannot be easily mixed with other methods, such as wet chemistry methods. Wu et al. [67] reported the combination of Al/CuO with an extra copper-based coordinate material Cu(NH_3_)_4_(NO_3_)_2_ using ARM. The resulting mixture is highly reactive and gas-generating due to the decomposition of Cu(NH_3_)_4_(NO_3_)_2_ during combustion. The size of the material was diminished to nanoscale by ARM to ensure a rapid reaction for its potential application in micro-airbag. Bekhouche et al. [68,69] utilized ARM to produce ternary NEMs of MgAl/CuO and MgAl/CuO/nitrocellulose, presenting a noticeable decreased activation energy with great heat of reaction and energetic performance.

## 5. Self-Assembled Nanoenergetic Composites

Self-assembly manifests when discrete components organize naturally into distinct structures at different length scales by precise interactions [70,71]. It is also possible to direct self-assembly by external agents/fields. These interactions are attributed to the underlying properties of the constituents composing the system or these interactions may be due to the application of external fields. As mentioned earlier, the rate of energy release during combustion reaction is dependent on factors such as arrangement, extent of interfacial contacts, shapes and sizes of fuels and oxidizers in NEMs. Consequently, various methods to self-assemble the fuels and the oxidizers in a defined manner have been reported in the literature. Self-assembled NEMs have been produced by exploiting and tailoring interactions between fuel and oxidizers. Various approaches reported so far include (i) electrostatic assembly [6,16,72], (ii) DNA-mediated self-assembly [17], (iii) polymer-mediated self-assembly [3,5] and (iv) functionalized graphene-directed self-assembly [18]. Such self-assembled NEMs show improved combustion properties by several orders over randomly mixed composites. Here the author highlights and discusses some key results from the published works with a focus on graphene-based NEMs. 

Preparation of self-assembled reactive Al-CuO microspheres with diameters of ~1–5 μm driven by electrostatic interactions has been previously reported [16]. Self-propagating combustion behavior is observed in microchannels upon ignition while the unassembled nanothermites does not show such behavior. Recently, self-assembled aggregates of Al nanoparticles and 2D sheets of MoO_3_ were reported, utilizing the electrostatic attraction between the fuel and the oxidizer [6]. The self-assembled composites exhibited excellent combustion characteristics (combustion wave speeds of up to 1730 ± 98.1 m/s peak pressures as high as 42.05 ± 1.86 MPa, and pressurization rates up to 3.49 ± 0.31 MPa/µs [6]. A monolayer coating of poly(4-vinylpyridine) (PVP) has been utilized to produce CuO nanorods/Al self-assembled energetic composites with the best burn rate of 2400 m/s (in comparison to 1900 m/s exhibited by unassembled composition) [3]. Monolayer coating is crucial to ensure reduced diffusion length for heat and mass transport. Self-assembled composites for other nanothermites have been produced following this approach [73,74].

In yet another method, self-assembled CuO nanorods around Al nanoparticles produced by a thin coating of poly(ethylene glycol) (PEG-400) exhibit excellent combustion performance [5]. Here, the hydroxyl (OH) and the methyl and methylene CH_n_ (n = 2, 3) functional groups present on the surface of CuO nanorods due to thin coating with PEG-400 are responsible for the observed self-assembly [5]. The superior combustion characteristics are credited to the improved conductive and convective heat transfer as a consequence of greater interfacial contacts between fuel and oxidizer and enhanced gas generation, respectively. 

In another work, DNA-mediated assembly of CuO and Al nanoparticles has been demonstrated, enabling micrometer-sized CuO/Al spheres [17]. DNA-directed assembly is essentially attributed to the coating of the constituents with single-stranded DNA molecules of complementary sequences and the resulting favorable interactions. Thermal analysis of this composite shows a 50 percent enhancement in heat of reaction (1800 J/g for self-assembled composites as opposed to 1200 J/g for randomly mixed composites. The onset temperature was reduced from 470 °C to 410 °C upon self-assembly. 

**Figure 3:**
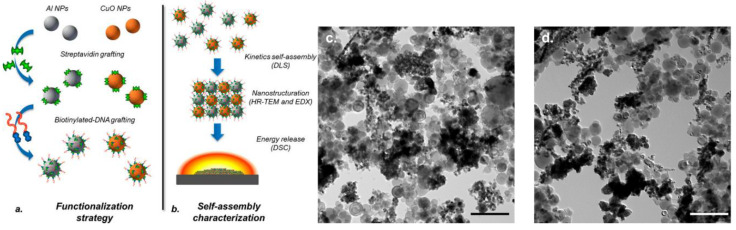
Schematic view of the (**a**) functionalization strategy and (**b**) multiscale characterization of biotinylated DNA grafted self-assembled Al-CuO nanocomposites in NaCl-based aqueous solution. HRTEM images of self-assembled (**c**) DNA_Al-CuO_35 mM and (**d**) DNA_Al-CuO_250 mM composites. Reprinted with permission from Théo Calais et al., *ACS Appl. Nano Mater.* 1 (9), (2018) 4716 [75]. Copyright © 2018 American Chemical Society.

Following this initial work, the authors further investigated the DNA-mediated self-assembly process through careful functionalization strategy and the use of optimized process parameters. The authors demonstrated a 240% increase in energy release of self-assembled DNA_Al-CuO mixtures compared to Al-CuO randomly mixed composite [75]. DNA mediated self-assembly process is quite elaborate as seen in Figure 3. Importantly, the entire self-assembly process was conducted in aqueous medium, which is a significant achievement. Interestingly, the authors report that the heat of the reaction of random Al-CuO mixtures produced in aqueous medium is higher by 40% than that of random mixtures of Al-CuO in hexane. The enhancement in energy release due to the DNA-mediated self-assembly has been specifically attributed to the functionalization of CuO and Al nanoparticles individually with Streptavidin prior to the mixing with biotinylated DNA, which allowed the preparation of homogeneously formed dense microstructure of the energetic composite as confirmed by HRTEM images (Figure 3). The kinetics of self-assembly were investigated with the presence of varying NaCl salt concentrations. A higher concentration of NaCl (say 250 mM) favors the formation of dendrite-based microstructure with reduced contacts between CuO and Al nanoparticles (Figure 3d). On the other hand, a lower concentration of NaCl favors the formation of dense and homogeneous Al-CuO composites (Figure 3c). Evidently, DNA_Al-CuO_35mM exhibited higher heat of reaction (2570 J/g) as opposed to a drastically reduced value of 610 J/g exhibited by DNA_Al-CuO_250mM. However, the scalability of this process will be a challenge given the meticulous nature of a multitude of process steps. The stability of the assembled composite also needs to be addressed carefully given the adverse reaction of Al nanoparticles with water. Meanwhile, the addition of such surface modification materials, which enables the self-assembly between fuel and oxidizer particles, adds dead mass to the final product and creates a barrier between nanoparticles during the reaction, lowering the final performance of the product.

Some indirect self-assembly methods are developed to avoid the introduction of inert additives. Fabrication of core–shell structured Al@CuO microparticles has been reported by Zhang et al., using thermal decomposition after assembling Cu(NH_3_)_2_^2+^ on the surface of Al particles [76]. Wang et al. applied the same method and fabricated nano-sized Al@CuO particles [77]. Compared to random Al-CuO mixtures, the core–shell Al@CuO exhibited lower onset and peak reaction temperature by 10–25 °C, a reduction in activation energy (237.6 kJ/mol vs. 258.4 kJ/mol), and shorter ignition (60 μs vs. 155 μs) and propagation delay (17 μs vs. 107 μs), which were caused by the enhanced surface contact and shortened average diffusion between fuel and oxidizer nanoparticles. The authors highlighted the process of the electrostatic-driven self-assembly process of Cu(NH_3_)_2_^2+^ ions on Al nanoparticles with negative surface charge, which can be finely controlled by the pH of the reaction environment. Cui et al. also reported an alcohol thermal treatment of Cu(OH)_2_ to produce a similar Al@CuO microspheres with enhanced combustion performance [78]. Similar coordinative-based methods can also be used to develop other Al@metal oxide core–shells such as Al@NiO [79].

A publication from Maini et al. describes the direct growth of Fe_3_O_4_ nanoparticles on Al nanoparticles to form a magnetic Al@Fe_3_O_4_ core–shell NEM [80]. The ferromagnetic Fe_3_O_4_ nanoparticles allow the particle to be delivered to target locations under the control of an external magnetic field, facilitating heat and pressure generation at hard-to-reach locations.

The direct formation of Al@metal oxide NEMs without the addition of surface treatment materials is indeed exciting for its high reactivity and flexibility. However, the high-temperature processing required during the fabrication may still negatively reduce the active percentage of Al core, and the safety issues of the nanocomposite during storage, transportation and utilization are still not addressed.

In the context of our discussion so far on self-assembly, it can be stated that graphene-based self-assembled EMs are most attractive for realizing superior combustion characteristics among various approaches reported in the literature as will be seen shortly [18,81]. However, given the relatively large number of articles published on employing graphene in energetic compositions, especially in the past decade, the focus of this review article is to examine the role of graphene and functionalized graphene sheets (FGS) in nanoenergetic formulations based on published literature. 

## 6. Role of Graphene in Energetic Composites

Among carbon-based materials, graphene and its derivatives such as graphene oxide (GO), reduced graphene oxide (rGO), nitrated graphene and halogenated graphene have been found to be very attractive as additives in energetic formulations. The derivates are often famously known as functionalized graphene sheets (FGS). Here, we attempt to provide a critical summary of research accomplishments reported in the literature to date and opportunities for future research on graphene-based energetic composites. Therefore, the authors expect that this section of the article will enable the readers to appreciate the role of FGS in improving combustion performance while reducing the sensitivity of NEMs significantly to electrostatic discharge, impact and friction. 

Among the carbon allotropes, graphene is a monolayer of carbon atoms in hexagonal lattice and has unique physicochemical properties such as large specific surface area (2630 m^2^/g), excellent electrical conductivity of (1.46 ± 0.82) × 10^6^ S/m, high thermal conductivity of 3000–5000 W/mK, outstanding mechanical properties with the fracture strength of 125 GPa and Young’s modulus of 1 TPa and carrier mobility of 2 × 10^5^ cm^2^/(Vs) [82,83,84,85]. With graphene and/or FGS as an additive in energetic formulations, it is natural to expect that these excellent characteristics enable not only high energy release rates but also ensure low risk of accidental ignition caused by electrostatic discharge, impact and friction [18,19,81,86,87]. Furthermore, the incorporation of graphene into energetic formulations is found to inhibit particle agglomeration and improve dispersibility, thereby providing better interfacial contacts. 

Shen et al. applied multi-layer graphene (MLG) as an additive to Al/CuO [88]. With only 1 wt.% of MLG addition, the energy release of as-produced Al-CuO-MLG was enhanced by 87.5 J/g. More importantly, the involvement of highly thermally conductive graphene in the reaction of Al/CuO improved the characteristics of reaction pathways of exothermic heterogeneous reactions. 

More than graphene, functionalized graphene sheets (FGS) have drawn greater attention in energetic formulations. FGS are also two-dimensional carbon sheets with high surface area, where the surface functionalities are molecularly tailored with the incorporation of energetic groups such as nitro (−NO_2_) and amine (−NH_2_). These materials have enabled the synthesis of energetic composites with tunable combustion performance with reduced sensitivity to external stimuli such as ESD, friction and impact. The presence of abundant oxygen-containing functional groups (carboxyl, hydroxyl, and epoxy groups) adhered on the surface and edge of GO is found to be beneficial for facilitating the formation of self-assembled energetic composites besides reducing the friction of the overall composite as a consequence of small friction coefficient of graphene. More often, GO is synthesized by the modified Hummers method. GO with increased oxygen content exhibits reduced electrical conductivity and therefore, it may not be as effective as pristine graphene in reducing the ESD. On the other hand, rGO prepared by the reduction of GO via a reduction reaction either with NaBH_4_, or N_2_H_4_ or by elevated temperature treatment under inert ambient, reveals better electrical conductivity and less functional groups in comparison with that of GO. However, GO has better solubility and dispersibility, which eases its processability. 

For EMs, the surface of GO can be modified with tailored functional groups such as -NO_2_, -NHNO_2_, and so on. The incorporation of nitrogen-containing functional groups is undoubtedly an attractive proposition for energetic formulations. The secondary decomposition reaction of these energetic groups is likely to add to the overall energy of the composite. In recent years, the synthesis of graphene-based organic compounds that exhibit improved thermal stability and good detonation performance has been reported [89]. Another related noteworthy mention is the use of FGS as a self-assembly directing agent to prepare highly reactive metal/metal oxide energetic mixtures with tunable combustion performance and reduced sensitivity [18,19,81,90]. The addition of FGS can also be utilized as a gasification material, enhancing the potential of EMs in gas-desiring applications such as propellants [91].

**Figure 4:**
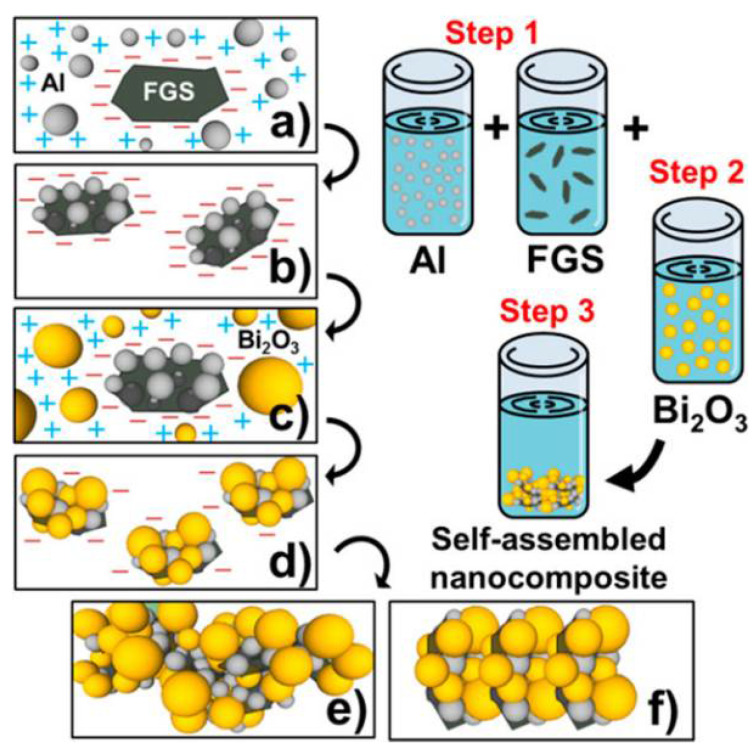
Representation of sequential steps of the self-assembly process. (**a**) Electrostatic attraction of Al to GO, (**b**) covalent bonding of GO/Al, (**c**) electrostatic binding of Bi_2_O_3_ to GO/Al, and (**d**–**f**) noncovalent assembly of Bi_2_O_3_ on GO/Al to form highly dense GO/Al/Bi_2_O_3_ macrostructures. Reprinted with permission from Thiruvengadathan et al., *Langmuir* 2014, 30, 22, 6556 [18]. Copyright © 2014 American Chemical Society.

**Figure 5:**
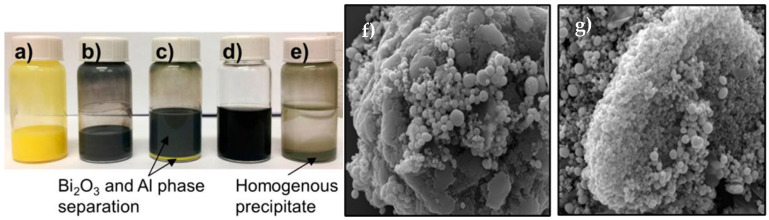
Homogeneous dispersions of (**a**) Bi_2_O_3_ (**b**) Al, (**c**) macroscopic phase separation in the absence of GO, (**d**) dispersions of GO, (**e**) precipitation of self-assembled Bi_2_O_3_/Al/GO composite, and (**f**,**g**) SEM images of self-assembled GO/Al/Bi_2_O_3_ nanocomposites in layered (**f**) and random (**g**) orientations. Reprinted with permission from Thiruvengadathan et al., *Langmuir* 2014, 30, 22, 6556 [18]. Copyright © 2014 American Chemical Society.

The functional groups incorporated on graphene upon controlled chemical functionalization are responsible for the observed hierarchical self-assembly of nanoscale oxidizer and fuel. Experimental evidence for the presence of both long-range electrostatic attractions followed by short-range covalent (between graphene oxide (GO) and Al) and noncovalent interactions (between GO/Al and Bi_2_O_3_) is distinctly illustrated in this work [18,81]. A schematic of the self-assembly process is shown in Figure 4. Directed self-assembly of Al and Bi_2_O_3_ nanoparticles on functionalized graphene sheets (FGS) was realized in a colloidal suspension phase that condensed into ultra-dense macrostructures (Figure 5). Importantly, the undesirable phase separation is evident in the absence of FGS. 

Significant increases in heat of reaction (up to 92% for optimized composition) were observed for self-assembled GO/Al/Bi_2_O_3_. For example, the heat of the reaction of self-assembled GO/Al/Bi_2_O_3_ material was 1421 ± 12 J/g, whereas it was only 739 ± 18 J/g for a randomly mixed Bi_2_O_3_/Al sample without the addition of GO. The enhancement is attributed to the benefits of self-assembly and the role of GO as an energetic reactant. The self-assembled composite exhibits excellent combustion characteristics (enhanced pressure generation from 60 to 200 MPa, reactivity from 3 to 16 MPa/µs, burn rate from 1.15 to 1.55 km/s, and specific impulse from 41 to 71 s). Importantly, the sensitivity to electrostatic discharge is shown to decrease significantly. Randomly mixed Al/Bi_2_O_3_ is highly sensitive to ESD and failed at 0.125 μJ. In contrast, the ESD sensitivity of self-assembled GO/Al/Bi_2_O_3_ composites decreased significantly. A maximum passing energy of 1.2 mJ was observed for composites with 5.0 wt.% GO. Enhanced combustion performance of FGS-based self-assembled nanothermites with reduced ESD augers well for their utility in a host of applications. Specifically, functionalized graphene plays the dual role as an additive (that augments the overall energy release) and as a self-assembly directing agent (that enhances the reaction rate). 

**Figure 6:**
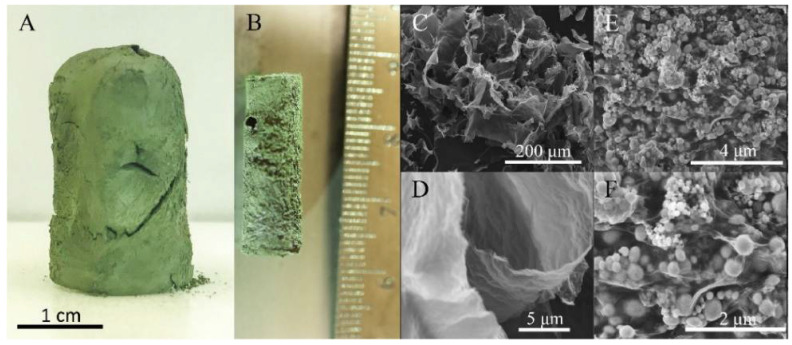
(**A**) Optical image of RGO/Al/Bi_2_O_3_ gel, (**B**) dimension of the gel reflected with a ruler in cm, SEM images of (**C**,**D**) neat RGO gel and (**E**,**F**) RGO/Al/Bi_2_O_3_ gel. Reprinted with permission from Anqi Wang et al., *Combustion and Flame* 2018, 196, 400 [19]. Copyright © 2018 Elsevier.

Wang et al. reported a novel method for the preparation of a macroscale energetic aerogel with a composition of RGO/Al/Bi_2_O_3_ gel that exhibits enhanced combustion characteristics with reduced ESD sensitivity (Figure 6) [19]. This energetic gel formulation is advantageous to overcome fundamental challenges such as particle aggregation due to sintering, stability, and inherent high sensitivity to ESD. This is exemplified by the following experimentally measured data. The macroscale gel exhibits an open burn rate of 960 ± 190 m/s in comparison to the neat Al/Bi_2_O_3_ nanothermite powder (without RGO) measured in a similar experimental arrangement that has a characteristic combustion wave speed of 460 m/s. The heat of the reaction in RGO/Al/Bi_2_O_3_ gel is 967 J/g, while it is only 739 ± 18 J/g in loose Al/Bi_2_O_3_ powder. Most importantly, the ESD sensitivity of the aerogel is decreased by three orders of magnitude in comparison to that of neat Al/Bi_2_O_3_ powder. The enhanced combustion performance exhibited by the macrogel presents undeniable evidence that chemical reduction and gelling procedures employed as part of the synthesis technique did not alter the inherent reactivity of Al and Bi_2_O_3_ nanoparticles besides sustaining the excellent interfacial contacts between them. The reduced ESD sensitivity is attributed to the presence of RGO, which offered an electrically conductive pathway. The unique synthesis methodology can be easily extended to other nanothermite systems too. Further research is essential to investigate the combustion performance of macroscale aerogel as a function of % TMD. Scalability and manufacturing methods need to be investigated, given the elaborate nature of preparation. 

**Figure 7:**
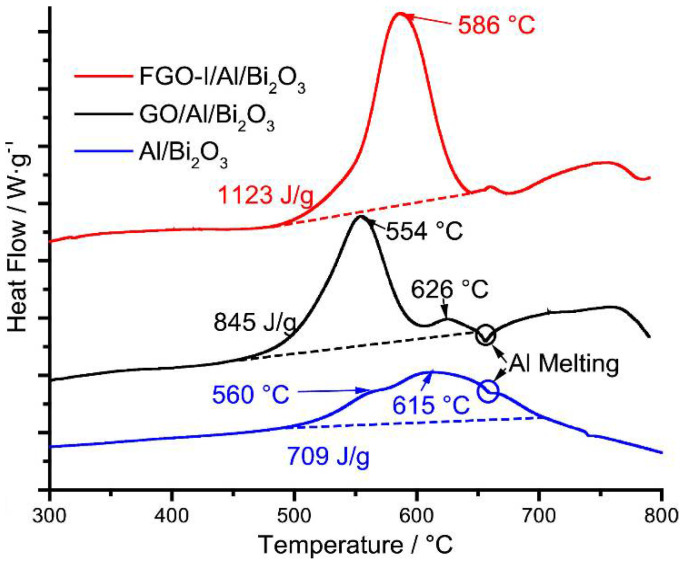
Plot shows the comparison of heat flow measurements of Al/Bi_2_O_3_, GO/Al/Bi_2_O_3_ and FGO-I/Al/Bi_2_O_3_ energetic composites. Reprinted with permission from Anqi Wang et al., *Combustion and Flame* 2020, 215, 324 [79]. Copyright © 2020 Elsevier.

In Section 1 of this article, the author discussed the presence of a native Al_2_O_3_ shell surrounding the metallic Al core in Al nanoparticles and the possibility of etching of Al_2_O_3_ shell with halogenated groups. Very recently, Wang et al. reported an alternative yet effective methodology to prepare fluorinated graphene oxide (FGO)/Al/Bi_2_O_3_ that utilizes functionalized graphene with fluorine and oxygen species [90]. GO prepared by modified Hummer’s method was exposed to xenon difluoride (XeF_2_) vapors in a custom-built chamber for varying durations in pulsed mode. The role of FGO as an energetic additive to Al nanoparticles and Al/Bi_2_O_3_ was investigated by a number of analytical methods including simultaneous thermogravimetry and differential scanning calorimetry (TGA-DSC), energy dispersive spectroscopy (EDS), T-jump mass spectrometry and Fourier-transform infrared (FTIR) spectroscopy. FTIR absorbance measurements revealed the replacement of hydroxyl (-OH) groups in C-OH and -COOH species by fluorine groups. The stability of FGO was evaluated by exposing it to 15% humidity at 25 °C (controlled environment in a desiccator). The hydrolysis reaction of acyl fluoride groups with free water in the atmosphere was noticed based on the decrease in the peak intensity of 1840 cm^−1^ (C=O stretching) peak along with a slight increase in the 3200–3700 cm^−1^ band. Thus, both unstable acyl fluoride groups and stable carbon-fluorine covalent/semi-ionic bonds were present in as-prepared FGO as evidenced by FTIR absorbance measurements as a function of storage time. Thermal stability tests performed using simultaneous TGA/DSC revealed a sharp decrease in fluorine content from 11 at. % to 0.4 at. % upon heating the sample to 600 °C. The key step in the preparation of FGO/Al/Bi_2_O_3_ composite requires using an ultrasonic bath to homogeneously mix the nanoscale constituents. Fluorine content decreased to 1–3.5% after ultrasonic treatment as found out from EDS measurement conducted on the dried sample. The ratio of fluorine to oxygen ratio dropped from 0.3 to 0.1 and the remaining fluorine is attributed to the presence of more stable C–F covalent bonds and semi-ionic bonds. Lesser amounts of fluorine may be enough to weaken the Al_2_O_3_ shell at lower temperatures, thereby exposing the active Al core to react with oxygen instantaneously and eventually, leading to solid-state combustion reaction. In fact, T-jump mass spectrometry measurements performed on FGO/Al/Bi_2_O_3_ composite provided evidence for this hypothesis. The measured heat of reaction for the FGO/Al/Bi_2_O_3_ sample is 1123 J/g in comparison to 709 J/g for randomly mixed sample Bi_2_O_3_/Al without FGO (Figure 7) Thus, it is evident FGO is an excellent energetic additive for realization of the following key goals, such as (i) to overcome the phase separation between the fuel and the oxidizer, (ii) to lower the onset of reaction temperature for favoring solid-state reactions and (iii) to enhance the overall energy release [90]. 

Jiang et al. utilized GO and GF (graphene fluoride) at the same time and fabricated an energetic Al/GO/GF without the usage of a separate oxidizer [92]. Experimental results exhibited higher reactivity of Al/GO/GF than Al/GO and Al/GF, and more impressively, higher than Al/GO/PTFE, where PTFE is involved as the main oxidizer. The splendid performance was then elucidated by reactive molecular dynamics. The involvement of GO highly provided energy release upon decomposition, while the high degree of dissociation, Al oxidation, and presence of reactive fluorine contributed by the GF acted synergistically to allow a more violent reaction to occur. Recently, Jiang et al. further introduced a fluorine-including multifunctional graphene-based additive, perfluoroalkyl-functionalized graphene oxide (CFGO), to NEM [93]. The fluorine, oxygen co-functionalized graphene utilized the advantages of both GO and GF, exhibiting superior reactivity. The existence of fluorine functional groups allowed the release of highly reactive fluorine gases, removing the Al_2_O_3_ shell of Al nanoparticles, while the oxygen functional group improved the homogeneity in mixing with Al nanoparticles and reduced the agglomeration of products. 

**Figure 8:**
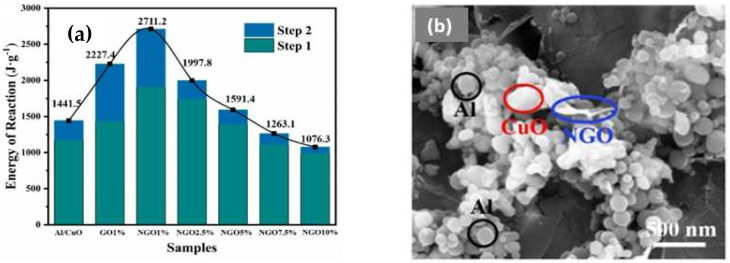
(**a**) Energy release of NGO/Al/CuO composites as a function of varying NGO content (**b**) SEM image of 3 wt.% NGO/Al/CuO composite shows the extent of interfacial contacts between Al and CuO and the spatial distribution of the three constituents. Reprinted with permission from Yueting Wang et al., *Combustion and Flame* 2021, 233, 111580 [82]. Copyright © 2020 Elsevier.

Wang et al. synthesized a novel NEM formulation using nitrated graphene oxide (NGO) as an energetic additive and investigated its catalytic effects on the combustion performance of Al/CuO as a function of NGO content up to 10 wt.% [94]. These NGO/Al/CuO composites prepared by wet chemical synthesis procedure showed enhanced and tunable combustion characteristics such as energy release and pressurization rate at reduced ESD sensitivity. The optimum composition that exhibits the best combustion performance is found to be with 1 wt.% NGO. While the energy release of neat Al/CuO nanothermites upon combustion is only 1441.5 J/g, the energy release of NGO/Al/CuO with 1 wt.% additive is 2711.2 J/g. Interestingly, the energy release of GO/Al/CuO with 1 wt.% GO is 2227.4 J/g. The heat of reaction decreases with increasing NGO content beyond 1 wt.% NGO as shown in Figure 8a. The thermal analysis of neat GO and neat NGO under an Ar environment at 10 °C/min provides another interesting observation. The onset and the peak temperature of exotherm decreases by 45 °C for neat NGO. The heat of reaction for NGO is higher than that of neat GO by 201 J/g. 

The ignition temperature was measured using a separate device built based on the T-Jump/TOF mass spectrometer. The ignition temperature of NGO/Al/CuO with 1 wt.% NGO is lowest at about 760 °C while it is 820 °C for neat Al/CuO. Coincidently, the pressurization rate determined from pressure–time characteristics is highest (about 32 kPa/μs) for this composite with 1 wt.% NGO. The burn rate of this composite with 1 wt.% NGO is 90.12 m/s, and this value is lower than 130.38 m/s measured for neat Al/CuO. As seen with the measured ESD values for GO-based nanothermites compositions, the sensitivity to ESD decreases with increasing NGO content in the NGO/Al/CuO composites. However, the combustion performance decreases with increasing NGO content beyond 1 wt.%. The authors attribute the decreasing performance to the increase in diffusion lengths between fuel and oxidizer with increasing NGO content in the composite. At the same time, the absence of diffraction peaks corresponding to the NGO phase in the X-ray diffractogram of NGO/Al/CuO composite even with 10 wt.% NGO confirms good exfoliation of NGO sheets, as the authors of this article have also stated. This observation is slightly perplexing. 

We believe that the amount of NO_2_ groups in GO is an important parameter that needs to be quantitatively determined. It is worth mentioning that the best combustion performance for GO-based Al/CuO formulation is achieved with 5 wt.% GO content while it is shown to be optimum at 1 wt.% NGO for NGO/Al/CuO. The high-resolution SEM image shown for this composite with 3 wt.% NGO reveals that there is phase separation between CuO and Al (Figure 8b) and therefore it is not surprising that the energetic performance decreases with increasing content of NGO beyond 1 wt.%. This decrease in combustion performance may be attributed to the deviation from the optimum ϕ value that defines the amounts of the oxidizer and the fuel used in the synthesis of these NGO/Al/CuO composites. Therefore, quantitative estimation of functional groups in GO and NGO will be the key to achieving further enhancement in combustion characteristics. The existence of -NO_2_ energetic groups in NGO is therefore attractive to realize better combustion performance. At the same time, the lubricating property of graphene and its analogues such as GO, and NGO may restrain the formation of hotspots and reduce the sensitivity.

Following the first work reported on GO/Al/Bi_2_O_3_ self-assembled nanothermite system [18], several similar NEM formulations but with other metal oxide systems such as Fe_2_O_3_, CuO and MoO_3_ have been reported. All such compositions exhibited enhanced reactivity and greater heat of reaction at reduced ESD sensitivity owing to improved homogeneous dispersion of constituents, thereby promoting better interfacial contacts between Al and metal oxides. For example, self-assembled rGO/Al/Fe_2_O_3_ synthesized using a combination of solution-phase synthesis and atomic layer deposition techniques exhibits an increased energy release by 50% in comparison to neat Al/Fe_2_O_3_ and random mixtures of rGO/Al/Fe_2_O_3_ with 4.8 wt.% rGO [95]. Very recently, Chen et al. reported the solution-phase assisted synthesis of GO/Al/p-CuO composite [96]. Here, p-CuO stands for porous copper oxide. The total heat of the reaction was best for the energetic composition with 5 wt.% GO content, and it was 2306.7 J/g. In all of these works, the optimum GO content is 5 wt.% independent of the metal oxide used in the energetic formulation. The reactivity reduces for composites prepared with higher content of GO.

**Figure 9:**
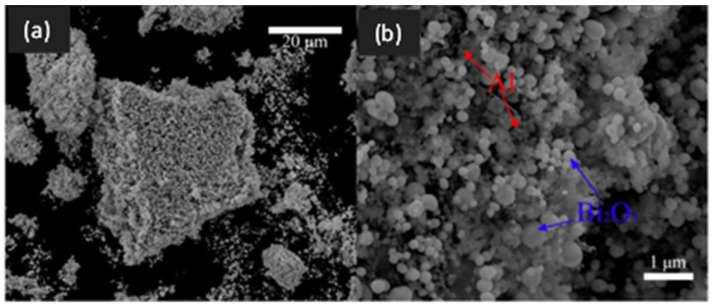
SEM image of I-rGO (7.5%)/Al/Bi_2_O_3_ composite recorded at (**a**) low magnification and (**b**) high magnification showing homogeneous mixing of fuel and oxidizer leading to dense microstructure. Reprinted with permission from Wang et al., *Nano Futures* 2020, 4(4), 045002 [97]. Copyright © 2020 IOP Publishing Ltd.

In yet another interesting work on graphene-based NEMs, the utilization of iodinated graphene (I-rGO) enables the self-assembly of fuel and oxidizer, thereby drastically inhibiting phase separation. Specifically, Wang et al. reported the synthesis and characterization of self-assembled I-rGO/Al/Bi_2_O_3_ composites that are found to exhibit enhanced combustion performance [97]. I-rGO sheets provided the scaffolds upon which Al and Bi_2_O_3_ were found to self-assemble first, followed by layer-by-layer assembly of decorated I-rGO/Al/Bi_2_O_3_ sheets as seen by the densification of decorated sheets in SEM images recorded at different magnifications (Figure 9). The improvement in combustion characteristics is attributed not only to the inhibition of phase separation of fuel and oxidizer but also to the ability of iodinated graphene to etch the alumina shell surrounding the metallic Al core, thereby reducing the diffusion lengths for mass and heat transport. 

**Figure 10:**
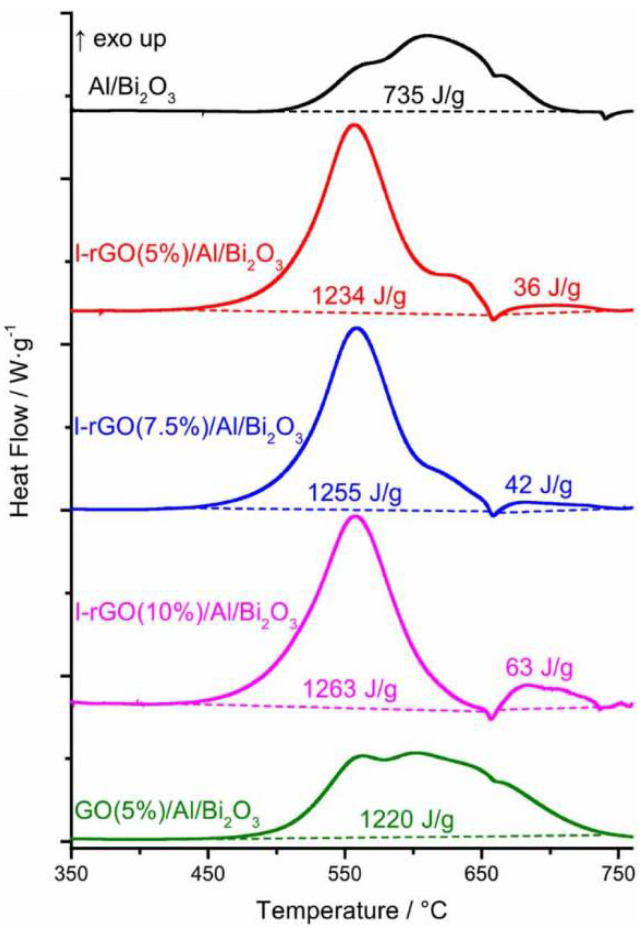
DSC thermograms showing the comparison of heat flow values of I-rGO-S/Al/Bi_2_O_3_, neat Al/Bi_2_O_3_ and GO/Al/Bi_2_O_3_ energetic formulations. Reprinted with permission from Wang et al., *Nano Futures* 2020, 4(4), 045002 [97]. Copyright © 2020 IOP Publishing Ltd.

Thermal analysis of I-rGO/Al/Bi_2_O_3_ showed a reduction in the onset and peak reaction temperatures besides a 70% enhancement in energy release in comparison to that of randomly mixed neat Al/Bi_2_O_3_ composite (Figure 10). Significantly, the etching of alumina shell by reactive iodine is evident from the fact that 95% of the total exothermic energy is released in the temperature range of 400–660 °C prior to the melting of Al. This confirms that the exothermic reaction occurred with metallic core Al in the solid state. ESD sensitivity of this I-rGO/Al/Bi_2_O_3_ is reduced by four orders of magnitude in comparison to neat Al/Bi_2_O_3_ without I-rGO. In summary, halogen-based oxidizers are attractive for NEMS primarily because of their high reactivity, enabling to damage of the alumina shell and thus facilitating a faster reaction with metallic aluminum. From the application point of view, Pantoya et al. experimentally showed the capability of Al/I_2_O_5_ nanothermite reaction to neutralize bacteria spores, which could be potentially used as biowarfare agents [98]. Therefore, it is more compelling to employ halogen-based graphene for its added value in guiding the self-assembly of fuel to oxidizer and vice versa. Also, comparing the role of FGO and I-rGO in NEMs, the latter is preferred for the following reasons though fluorine has higher reactivity than iodine. Firstly, the onset initiation temperature of FGO/Al/Bi_2_O_3_ is 50 °C higher than that of I-rGO/Al/Bi_2_O_3_. Secondly, the heat of the reaction of FGO/Al/Bi_2_O_3_ (1123 J/g) is lower than that of I-rGO/Al/Bi_2_O_3_ (1234 J/g). These two facts are true for FGO/Al and I-rGO/Al composites also without the addition of Bi_2_O_3_. 

**Figure 11:**
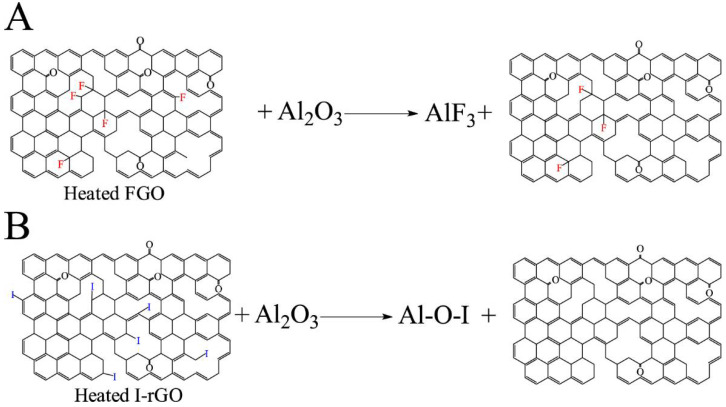
Schematic pathways for the reaction of (**A**) FGO and (**B**) I-rGO with Al_2_O_3_ shell of Al nanoparticles. It is evident that iodine from the weak C-I bond is released to enable a complete reaction with Al_2_O_3_, C-F bond is relatively stronger, making it difficult for fluorine to be released easily during heating for a complete reaction with the Al_2_O_3_ shell. Reprinted with permission from Wang et al., *Nano Futures* 2020, 4(4), 045002 [97]. Copyright © 2020 IOP Publishing Ltd.

These differences in the experimentally measured observations between FGO/Al/Bi_2_O_3_ and I-rGO/Al/Bi_2_O_3_ are attributed to the following: (i) amount of halogen atoms in functionalized graphene, (ii) nature of bonding of halogen atoms to functionalized graphene, (iii) decomposition of acyl fluoride groups during the synthesis of FGO/Al/Bi_2_O_3_ composites, and (iv) loss of 95% fluorine prior to thermite reaction as opposed to release of iodine during thermite reaction. It is also envisioned that fluorine in functionalized graphene is present as semi-ionic and covalent C-F stable bonds. The reaction schemes of FGO and I-rGO with an Al_2_O_3_ shell are shown in Figure 11. These reasons make I-rGO a more attractive choice than FGO for energetic formulations. 

In another work, Wang et al. demonstrated the usefulness of employing spinel MCo_2_O_4_ (M = Cu, Mg, Zn and Ni) as oxidizers in novel nanothermite compositions with Al as fuel using GO as a self-assembly directing agent [99]. Among the various compositions investigated in this work, CuCo_2_O_4_/GO/Al prepared at a ϕ value of 1.5 with 2.5 wt.% GO content exhibits the highest heat of reaction of 3422.0 J/g. The pressure–time characteristic of this composition also shows the shortest pressure rise time (27.18 ms) and higher peak pressure (6.03 MPa). Importantly, the ESD sensitivity of CuCo_2_O_4_/GO/Al is significantly reduced. The extent of self-assembly in these compositions needs to be quantitatively measured. From this discussion, it is clearly evident from several works that the combustion performance of graphene-based nanothermite compositions at reduced ESD sensitivity is improved significantly attributed to the self-assembly of fuel and oxidizer directed by FGS.

In contrast, Song et al. reported that the presence of GO in Al/MnO_2_ nanothermite composition totally hinders the self-propagating combustion reaction [100]. The authors attribute this unexpected observation to the higher stability and barrier properties of GO, consequently reducing the heat and mass transfer of materials. More specifically, the thermal decomposition of MnO_2_ at a lower temperature in the presence of GO prior to the onset of thermite reaction around 500 °C as seen from thermal analysis experiments and therefore loss of oxygen is cited as the primary reason. The authors cite the reduced mass transport of fuel and oxidizer due to the two-dimensional structure of GO. The authors further note that the use of GO as a flame-retardant additive in polystyrene- and polydimethylsiloxane-based compositions has been well reported in the literature. Thus, it is interesting and puzzling to note that this kind of suppressed combustion behavior seen in the Al/MnO_2_/GO nanothermite system is in total contrast to the enhanced combustion characteristics exhibited in other nanothermite systems. Given that the use of GO as an energetic additive and also as a self-assembly directing agent has worked wonders with all other nanothermite systems in enhancing the combustion performance, further work may be necessary to pay attention to the synthesis and characterization of GO. Particularly, the number of layers of GO, the amount of various functional groups present in GO and the size distribution of GO should be determined to understand the hindering mechanism of GO in the combustion propagation of Al/MnO_2_/GO. It may also be necessary to investigate if GO was reduced to rGO during its reaction.

## 7. Application Development of NEMs

In the following subsections, we review the key developments in the utility of nanoenergetic formulations in some applications. 

### 7.1. Energetic Microdevices

There has been a sustained interest in the development of highly accurate, dependable, customizable and safe energetic microdevices for advanced pyrotechnics. Additive manufacturing, especially direct ink writing, using energetic ink formulations as active material has been utilized to develop such devices. However, nanoparticles themselves cannot bind together and form 3D devices with stable structures, and additives are required to allow the three-dimensional structure to form and the nanoparticles to assemble on [101]. Traditionally, polymers are used as the main structural additive during the AM process of NEMs, such as fluororubber [102], HPMC [103], and PVDF [104]. The emulsification effect of polymer adjusts the rheology of the ink and provides structure strength between polymer chains after the material is dried post printing. GO is also reported as a gasification additive along with structural polymers [105]. The usage of the polymer is simple and efficient and allows AM of NEMs but may also negatively affect the reaction due to the dead mass and slow decomposition process.

Curing of the polymer post printing is the critical step in the direct writing process and particularly, curing time is a key parameter. The conventional method of employing adhesive is inefficient in the sense that the curing time is pretty long. Very recently, Kong et al. employed a UV-curing method to efficiently cure CL-20-based explosive ink in a groove of 1.0 × 1.0 × 100 mm, realizing a density of 1.612 g/cm^3^. The molded sample revealed a uniform distribution of explosive and binder and the curing time is reported to be just 8 min. Importantly, a detonation velocity of 7129 m/s is realized in a 1.0 × 1.0 × 100 mm^3^ device and at the same time, the sensitivity of this CL-20-based UV-curing explosive ink formulation to impact is shown to be drastically reduced [106]. This development is indeed promising and simultaneously, one can take advantage of 3D printing technology to form patterned energetic microdevices.

**Figure 12:**
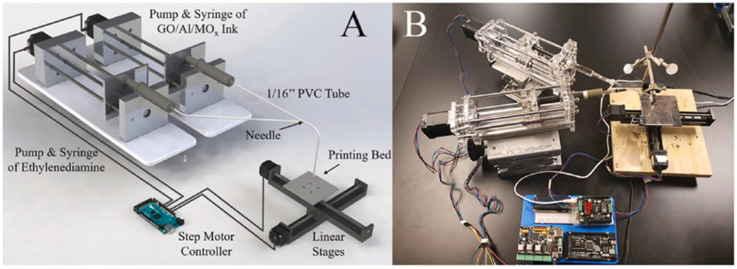
Schematic (**A**) and actual photograph (**B**) of the printing setup. Reprinted with permission from Anqi Wang et al., *Carbon* 2024, 216, 118596 [107]. Copyright © 2024 Elsevier.

Recently, Wang et al. reported a rGO/Al/metal oxide (MO_x_) aerogel fabricated by a polymer-free AM method based on the chemical reduction of GO [107]. A schematic of the experimental setup fabricated by the authors and an actual photograph of the setup is shown in Figure 12. GO/Al/MO_x_ ink in propylene carbonate was mixed in line with ethylenediamine, which reduced GO to rGO and formed an interconnected 3D structure as the skeleton. After drying, the final rGO/Al/MO_x_ aerogel presented a highly porous structure with Al and MO_x_ nanoparticles wrapped in rGO sheets. The combustion speed reached up to 6 m/s and could be tailored by adjusting the percentage of GO and NEM in the original ink. The high thermal conductivity of the rGO skeleton promoted the heat transfer in the pre-heating zone and accelerated the propagation of the combustion. The broader adoption of various AM methods in the fabrication of NEMs will significantly boost the applications of these materials to meet specific requirements on different occasions, such as micro-/nano-satellite propulsion [107], end-of-life self-destruction [108], customizable welding [109], etc.

Micro electro-mechanical systems (MEMS) devices have been considered a key part of potential NEM applications in microdevices with many publications in the past decades [13]. Specifically, the utilization of highly reactive NEMs fulfills the interest of miniaturization of modern pyrometric devices. Li et al. developed a Co(OH)F@Al nanobelt array specifically for fabrication on Si wafers and other substrates for MEMS devices [110]. The hydrothermal synthesis of the process of the material is highly compatible with the MEMS production process and provides great energetic performance due to the existence of both oxygen and fluorine in the oxidizer. More recently, Liu et al. also developed a nano [Cu(BODN) ·5H_2_O]@Al energetic film on a copper substrate using in situ synthesis and drop casting [111]. Thanks to the synthesis reaction between Cu(OH)_2_ and the porous supramolecular structure of BODN, the morphology and composition can be simply adjusted by reaction time and concentration to achieve adjustable reactivity of the final product. The as-produced energetic [Cu(BODN) ·5H_2_O]@Al films achieved an outstanding energy release of near 2000 J/g, indicating a great possibility of its integration as pyrotechnic material in MEMS ignition chips.

In yet another effort to realize a miniaturized pyrotechnic device, inkjet printing methodology has been suitably applied to print a glycidyl azide polymer/nitrocotton/3,4-dinitrofurazanofuroxan ink with a layer thickness of less than 10 μm and the printed sample exhibits high strength, high detonation speed and small critical dimension [112]. The molding density is estimated to be 1.773 g/cm^3^. Importantly, the critical detonation thickness is found to be 15 μm and the printed sample exhibits a detonation velocity of 8686 m/s. This study augers well for the application of this material as well for the employment of inkjet printing technology to develop micro initiating charges for use in MEMS devices.

NEM-based microdevices are also desirable in modern ammunition, where MEMS-based pyrotechnics are composed of an initiator, a safe and arming device, and a lead charge. Such an MEMS-based device is compact and attractive. The initiator is usually a nanothermite coating on a NiCr alloy foil or Pt pattern. A safe and arming device is kept between the initiator and the lead charge. The entire device is designed and fabricated in such a manner that it allows normal detonation in the arming condition and prevents accidental detonation in the safe condition. It is evident that the key to guaranteeing the safety and reliability of the ammunition system lies in the design of a safe and arming device. In this regard, Wang et al. recently reported the fabrication and characterization of an MEMS pyrotechnic with a double-layer barrier safe and arming device with a dimension of 13.4 × 8.5 × 5.2 mm^3^ [113]. The initiator is a NiCr bridge foil covered with an Al/CuO energetic film. The lead charge is lead styphnate. The safe and arming device consists of four semi-circular barriers, guided by V-shape electro-thermal actuators to enable active management of ignition conditions with rapid response. Such a design has been shown to support normal detonation in the arming condition and to prevent accidental detonation in the safe condition. 

### 7.2. Nanoenergetic Additives for Propellants

Studies on the incorporation of graphene and its derivatives as additives to catalyze combustion reactions in solid propellant formulations with improved mechanical strength are well reported in the literature. The inclusion of graphite and carbon black in energetic formulations is well known. Graphite with high thermal conductivity and efficient lubricity is a known desensitizer. The inclusion of carbon black is found to reduce ignition delays and decrease sensitivity. The inclusion of graphene in 1,3,5-triamino-2,4,6-trinitrobenzene (TATB) is found to be beneficial to realize a graphene–TATB composite with a combustion velocity of 2.40 km/s and a detonation pressure of 10.5 GPa. Furthermore, it is shown that one can produce such a composite with varying proportions of graphene and TATB can exhibit tunable characteristics such as packing density, thermal stability, sensitivity and detonation [114]. Encapsulation of explosives such as cyclotetramethylenetetranitramine (HMX) by coating with GO is found to decrease the impact sensitivity significantly (by 90%) and friction probability by 68%. Importantly, only 2 wt.% of GO is needed to achieve this drastic reduction in sensitivity [115]. Interestingly, the addition of Viton, (which is an elastomer) to GO/TATB energetic composite reduces the impact and the shock sensitivity of the resulting formulation. In another work, CL-20 was grown on graphene foam, and the resulting energetic composite exhibited reduced sensitivity to electrostatic discharge, impact and friction while reasonably sustaining the detonation velocity and pressure [116]. In short, the insertion of graphene and its derivatives in conventional explosive formulations is found to decrease the decomposition temperature and activation energy and reduce the sensitivity to external stimuli. This observation is independent of the preparation method. Total energy release and rate of energy release can be optimized by mixing controlled amounts of graphene and its derivatives in energetic formulations. 

The presence of graphene and its derivates in energetic formulations can control the ignition and combustion properties. Ignition is normally initiated by external stimuli such as electrothermal or photothermal means. The time taken from the onset of external stimulation to sustainable ignition defines the ignition delay time. More often, it is desired to have shorter delay times. Commonly, ignition delay is influenced by the chemical composition of energetic formulations, their packing density, and the presence/absence of a catalyst. Among various catalysts, GO is attractive to catalyze the thermolysis of energetic formulations as well as to augment the total energy release and the reaction rate via participation in the decomposition reaction. Consequently, graphene-based energetic formulations exhibit an ignition threshold and an increased combustion rate. This observation has been well demonstrated by Sabourin et al. in their work on the combustion of nitromethane (NM) with the addition of colloidal particles of FGS and metal hydroxides [86]. The burn rate of energetic formulation with 0.2% GO was 175% over pure NM without GO. The decomposition mechanism was attributed to the vacancy defects within the plane of the FGS. This results in an exchange of protons or oxygens between the oxygen-containing functional groups and nitromethane and its derivatives. In another work, the incorporation of GO in nitrocellulose enabled a combustion reaction upon initiation by laser (2 W, 50 ms, 1064 nm, 2 mm spot size) [117]. On the other hand, the combustion of neat nitrocellulose could not be initiated with the same laser with identical parameters. This observation shows the catalytic activity of GO to initiate photoignition of the NM/GO composite. 

Wang et al. employed the sol–gel method to impregnate AP into 3D porous graphene aerogels (GA) with the microscopic image revealing the surface coverage of GA by AP crystals [118]. The thermolysis rate and total heat release of the resulting AP/GA composite significantly increased, indicating the participation of GA in the oxidation reaction as a fuel. Burning rate is an important metric that determines the efficacy of solid propellants in particular. In several works, it has been experimentally confirmed that graphene and its derivates as additives in propellant formulations have increased the burning rate. The improvement in the burning rate of solid propellants is attributed to the continuous thermal conduction path facilitated by the 3D porous structure of graphene foams. 

Apart from graphene and its derivatives, metals and metal oxides have been successfully used as additives to enhance the burning rate of propellants. On the other hand, the addition of graphene in energetic formulations is shown to reduce sensitivity to ESD, impact, and friction. Therefore, it is expected that the presence of both graphene and metal/metal oxides together in propellants can produce a synergistic effect. For example, it has been shown that ignition and combustion properties of Al/GO composite can be tailored with the presence of GO facilitating optical absorption and thus ignition [119]. Another study on the catalytic application of Ni/graphene for the thermal decomposition of ammonium perchlorate (AP) revealed the disappearance of low-temperature decomposition peak and the decrease in decomposition temperature of high-temperature peak by 97.3 °C in a composite of Ni/graphene/AP [120]. Other compositions of M/FGS (with M = Cu, Au, Mn or bimetallic like Ni/Mn combination) as catalysts. have also been investigated and reported in the literature [121,122,123].

Nanoscale metal oxides such as CuO, Fe_2_O_3_, Bi_2_O_3_, NiO, Cr_2_O_3_, and MnO_2_ have been used as catalysts in propellant formulations in an effort to enhance the burning rate, to reduce the ignition delay, to decrease the pressure exponent and to promote combustion stability [124]. The biggest issue with the use of nanoparticles is aggregation, therefore hindering their efficiency in realizing the above-mentioned objectives. On the other hand, graphene with a high surface area promotes uniform dispersion and homogeneous distribution of metal oxide nanoparticles and this has been experimentally investigated and demonstrated in the preparation of graphene/metal oxide composites using a number of synthesis methods such as chemical precipitation, electrostatic self-assembly, hydrothermal/solvothermal and sol–gel methods. These graphene-based metal oxide composites have been evaluated for their efficacy in catalyzing the decomposition reactions of propellants. For example, incorporating only 2% CuO/GO prepared using chemical precipitation in AP lowers not only the thermal decomposition temperature from 414 °C to 315 °C, but also increases the heat output from 590 to 1347 J/g [125]. The application of mixed metal oxide–graphene such as Cu_2_O-PbO/GO and CuO-Bi_2_O_3_/GO in propellants is shown to enhance the combustion performance of DB and CMDB propellants due to the dispersion effect of GO and synergistic catalytic effect of both the metal oxides and the GO [126].

In another work, 5 wt.% of Mn_3_O_4_/rGO composites (previously synthesized solvothermal method) introduced in propellant formulation reduces the high-temperature decomposition temperature of AP by 141.9 °C [127]. Employing the solvothermal method, maghemite (g-Fe_2_O_3_) was in situ assembled on rGO to form g-Fe_2_O_3_/rGO composites and was then tested on the thermal decomposition of CL-20. The decomposition temperature of CL-20 was lowered by 6.93 °C and the activation energy was also lowered by 32.34 kJ/mol [128]. In general, a combination of metal oxide and graphene performs better as a catalyst in tailoring the combustion characteristics of propellants than the ones that use only either metal oxide or graphene or its derivatives. 

The GO-MgFe_2_O_4_ composite prepared by electrostatic self-assembly shows better catalytic activity on the thermal decomposition of AP than GO/MgFe_2_O_4_ prepared by ultrasonic mixing [129]. Similarly, Fe_2_O_3_/graphene aerogel prepared by the sol–gel method shows excellent catalytic activity on the thermal decomposition of AP. The two exothermic decomposition processes normally seen in AP thermal analysis disappeared with increasing content of Fe_2_O_3_/graphene aerogel and only a single exothermic process at a reduced decomposition temperature was observed together with larger heat release [130]. In all of the above examples, the catalytic activity of graphene-based neat and composite materials in lowering the activation energy, decreasing the decomposition temperature, increasing the total energy release (in comparison to the combustion of propellants without catalysts) and stabilizing the combustion process is clearly evidenced through meticulous experiments. 

### 7.3. Hybrid Energetic Formulations with Reduced ESD Sensitivity

One of the major challenges that is being persistently faced in employing neat nanothermites in practical applications is their high sensitivity to ESD. The combustion of neat nanothermites does not generate enough gas. This limits its application ability, for example, in the microthrusters. Zhang et al. reported a systematic study on hybrid energetic formulations composed of nanothermites (either Al/CuO or Al/MoO_3_) and CL-20 in varying proportions in an effort to realize enhanced impulse and specific impulse at a reduced sensitivity to ESD [131]. In the case of the Al/CuO/CL-20 system, the highest impulse value of 206.66 μN-s and the highest specific impulse value of 6.1 s was recorded with 10 wt.% CL-20. In the case of the Al/MoO_3_/CL-20 system, the highest impulse value of 1225.34 μN-s and the highest specific impulse value of 6.1 s were recorded with 30 wt.% CL-20. The ESD sensitivity of these compositions was evaluated by determining the energy for a 50% probability of ignition (E_50_). In comparison to extremely low E_50_ values of 0.24 ± 0.06 mJ and 0.28 ± 0.07 mJ measured for neat nanothermites, the values of E_50_ are 10.50 ± 1.34 mJ and 9.21 ± 1.45 mJ for Al/CuO/10 wt.% CL-20 and Al/MoO_3_/30 wt.% CL-20, respectively. Evidently, hybrid EMs are less sensitive to ESD, and therefore, it is an attractive proposition to employ hybrid EMs in applications. 

### 7.4. Nanoenergetic Materials for Self-Destruct Devices

Another interesting potential application of NEM is in self-destruct devices, thanks to the rapid reaction rate, extremely high temperature and pressure generated during its combustion. The self-destruction of multiple devices, such as defense equipment, medical devices, and personal financial devices, can effectively prevent data leakage or key apparatus stolen by undesired people or organizations. Yang et al. developed BiOBr/Al/Bi_2_O_3_ and fluorinated graphene/Al/CuBi_2_O_4_ for self-destructive microchips [132,133]. The combustion of NEM can be triggered simply by a hot wire integrated within the microchip, destructing the chip completely or simply de-functionalizing a key electronic component on the microchip to prevent further usage. The high heat and gas released from the reaction could complete the destruction process within 1 s. A similar observation was also published by Pandey et al. [134] that self-assembled Al/CuO coated on the Si chip can effectively destroy the target chip via resistive heating or an electrical spark.

### 7.5. Microthrusters

Microthrusters are small-sized propulsion devices used mostly in spacecraft and satellites. Microthrusters facilitate fine guidance, precision maneuvers and adjustments over movement, orientation, and placement in space. Dimensions of microthrusters are typically a few centimeters. Among the various types of microthrusters, thrusters employing NEMs as propellants are very popular. Specifically, nanothermites have been found to be very attractive owing to their ability to satisfy a number of requirements for microthrusters. Nanothermites exhibit high energy density, high reactivity, fast ignition times, less residue post-combustion, and tailorable combustion performance. Furthermore, only very small amounts of nanothermites are needed to realize desirable thrust characteristics primarily due to their high energy density. Therefore, microthrusters employing nanothermites are structurally compact and lightweight. The two main metrics used to define the performance of NEMs for microthruster applications are specific impulse I_sp_ and volumetric impulse I_SV_ as defined by the following equations,

$$I_{SP}=\frac{1}{W_{p}}\int_{0}^{t}Fdt$$

$$I_{SV}=\frac{1}{V_{p}}\int_{0}^{t}Fdt$$

where W_p_, V_p_, t, and F are the weight of the nanothermite/propellant, the volume of the the nanothermite charge, the burn duration and the thrust, respectively. 

Microthrusters employing neat Al/CuO and neat Al/Bi_2_O_3_ nanothermite systems have produced thrusts with specific impulses of 20–25 s and 41 s, respectively [135,136]. Although these values appear to be much smaller in magnitude in comparison to traditional propellants used in large rocket engines (greater than 250 s), the inherent energy losses associated with miniaturized motors (such as those used in microthruster) force the I_sp_ values to generally remain below 20 s. Therefore, these values are quite good. In general, the key to realizing greater I_sp_ lies in increased gas generation during combustion and in overcoming incomplete combustion. The incomplete combustion arises primarily due to the phase separation of the fuel (Al nanoparticles) and the oxidizer (metal oxide). Combustion of neat metal/metal oxide nanothermite systems does not produce enough pressure to realize higher thrust/force. Gas generation can be enhanced via the incorporation of an energetic additive that decomposes into a gaseous state completely during combustion. Therefore, Staley et al. exemplified the use of nitrocellulose (NC) as an energetic additive and binder in the Al/Bi_2_O_3_ system prepared in varying ϕ values [136]. In this work, the nitrocellulose content was varied from 0 to 5 wt.%. The value of I_sp_ is found to be optimum, and it is 63.2 s at an equivalence ratio of 1.6 for the composition of Al/Bi_2_O_3_/2.5(wt.%) NC. The significant improvement in the I_sp_ value in comparison to neat Al/Bi_2_O_3_ is attributed to the inhibition of phase separation between Al and Bi_2_O_3_ system, thereby enabling complete combustion. Decomposition of NC during combustion reaction also enhanced the peak pressure and thus higher thrust. Increasing the NC to 5 wt.% in this composition did not help to increase the I_sp_ value. This is attributed to the increased diffusion barrier between the fuel and the oxidizer, thereby slowing down the combustion reaction which results in lower thrust. Though increasing the burn duration is expected to enhance the I_sp_ value, the higher energy loss through the engine walls results in a significant reduction in thrust, leading to lower I_sp_. Thus, it is evident that there is a tradeoff between burn time and thrust generated. Furthermore, the authors performed the stability tests of Al/Bi_2_O_3_/NC loaded miniaturized thrusters by subjecting them to high-g launch using a gas accelerator system. These tests were conducted to understand the thruster performance post high-g events. Specifically, accelerations in the range of 50 to 90 kg were used in these tests. Following the high-g launch tests, the thruster motors were detached from the integrated test articles and were physically examined to check for their survivability. Then, the force–time characteristics were determined on those survived motors. Interestingly, the values of average thrust, burn duration and I_sp_ did not change significantly post high-g launch in comparison to those of control samples (those that were not subjected to high-g acceleration tests). These observations were true for nanothermite systems with and without NC. These tests demonstrated the usefulness of nanothermite-based propellants for microthrusters applications with high reliability and reproducible characteristics. 

We have previously discussed the efficacy of GO as a self-assembly directing agent and as an energetic additive in promoting the combustion characteristics of GO/Al/Bi_2_O_3_. Additionally, the significant enhancement seen in peak pressure and pressurization rate of this composition in comparison to those of neat Al/Bi_2_O_3_ is, in particular, an attractive proposition for microthrusters. Thiruvengadathan et al. investigated the force–time characteristics of GO/Al/Bi_2_O_3_ as a function of GO content (0 to 5 wt.%) [81]. Importantly, the value of I_sp_ increased from 41 s measured for the neat Al/Bi_2_O_3_ system to 71 s measured for 5 wt.% GO/Al/Bi_2_O_3_ system. The improvement in thrust characteristics has been attributed to the complete combustion of NEMs and increased gas production owing to the thermal decomposition of GO. Also, the sensitivity of Al/Bi_2_O_3_ to ESD is lowered by four orders of magnitude upon incorporation of GO. Still, the ESD energy value measured for 5%GO/Al/Bi_2_O_3_ is 1.2 mJ, which indicates the need to decrease the sensitivity further. 

**Figure 13:**
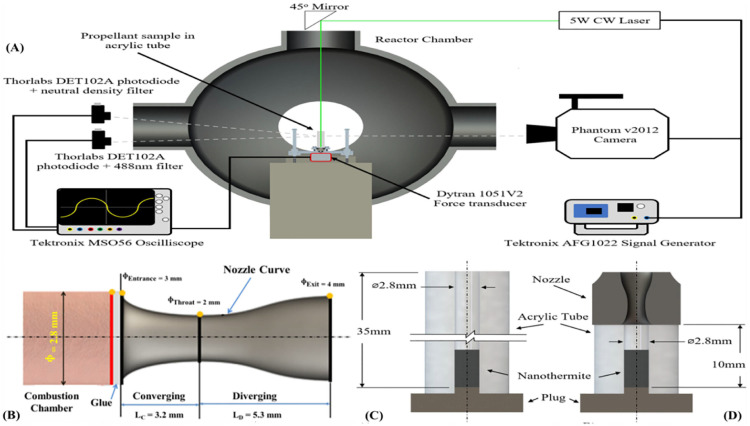
Schematic of (**A**) experimental setup (**B**) nozzle (**C**) Dimension of open small-scale test motor (STM) (**D**) Dimension of converging-diverging STM. Reprinted with permission from Fahd et al., *Combustion and Flame*, 2021, 232, 111527 [91]. Copyright © 2021 Elsevier.

Following this work, there have been a number of reports examining the ways to improve the thrust characteristics of NEMs and to decrease the ESD sensitivity to enable safe functionality. The traditional definition of nanothermites as a mixture of metal and metal oxide has been later extended to include mixtures of Al nanoparticles and oxygenated/fluorinated oxidizers in an effort to enhance the combustion performance, in particular, to realize gas generation. This suits well for microthruster applications [137]. Specifically, the higher atomic oxygen content in oxygenated salts is a desirable proposition to enhance the reactivity through enhanced gas generation during combustion and thereby supporting the convection mechanism. Interestingly, Wen et al. reported the synthesis and characterization of a quaternary NC/GO/Al/KClO_4_ nanothermite using facile electrospinning [91]. Evidently, the choice and inclusion of NC and GO as gas-generating and energetic additives appear to be based on previous works reported in the literature [81,136,138]. The choice of KClO_4_ as the oxidizer in NEMs is attributed to its high oxygen content, vigorous oxidizing nature, and heat-releasing decomposition. The authors of this work studied the thrust characteristics using a small-scale test motor with and without a converging and diverging nozzle, supported by laser ignition and high-speed camera imaging, as shown in Figure 13. 

The study was conducted on NC/GO/Al/KClO_4_ compositions as a function of varying NC content from 0 to 10 wt.% in increments of 2.5 wt.% while the GO content was kept constant at 5 wt.%. The ϕ value of nano Al and KClO_4_ was kept constant at 0.8. Among all compositions studied in this work, 5%NC/GO/Al/KClO_4_ exhibited the best thrust performance in terms of I_sp_ value and it is a remarkably very high value of 203.2 s, which is nearly three times in comparison to the value realized with either NC or GO-based Al/Bi_2_O_3_ nanothermite system. Therefore, the presence of both GO and NC together in the energetic composition is indeed a unique value addition for microthruster application. It would have been good to determine the ESD sensitivity of 5% NC/GO/Al/KClO_4_ samples. Surprisingly, Fahd et al. [139] reported a study on the thrust characteristics of GO/Al/KClO_4_ two years later than the similar work on NC/GO/Al/KClO_4_ reported by Wen et al. [91]. However, this study on GO/Al/KClO_4_ emphasizes the crucial role played by GO in enhancing the thrust characteristics. The optimum I_sp_ value of 5%GO/Al/KClO_4_ composition was 135.20 s at 20%TMD, an improvement of 57% compared to a value of 85.88 s shown by neat Al/KClO_4_ samples. Comparing the works of Fahd et al. and Wen et al., the synergistic role of both GO and NC in enhancing the thrust characteristics of NC/GO/Al/KClO_4_ is clearly evident. 

In yet another recent work, Zhang et al. investigated an interesting composition of Al/M_X_O_Y_ @CL-20 where M_X_O_Y_ stands for either CuO or MoO_3_ [131]. Among the compositions tested as a function of CL-20 content, Al/MoO_3_@30%CL-20 exhibits an optimum I_sp_ value of 32.75 ± 7.28 s. On the other hand, Al/CuO@10%CL-20 exhibits an I_sp_ value of 6.10 ± 0.64 s. Though these values were far lesser than that exhibited by 5% NC/GO/Al/KClO_4_ reported by Wen et al., the ESD sensitivity of Al/M_X_O_Y_ @CL-20 was greatly reduced, thus increasing the safety aspects immensely. The ESD energy for 50% probability of ignition (E_50_) of Al/CuO@10%CL-20 was 10.50 ± 1.34 mJ while that of neat Al/CuO was 0.24 ± 0.06 mJ. Similarly, the E_50_ value of Al/MoO_3_@30% CL-20 was 9.21 ± 1.45 mJ in comparison to a value of 0.28 ± 0.07 mJ exhibited by neat Al/MoO_3_. In a related study, Martirosyan et al. reported a study on the design and evaluation of microthruster and thruster arrays using an additive three-dimensional printing process, which augers well from the viewpoints of structural design, propellants synthesis, system-level integration, sensing and controlling electronics [140]. The thrust characteristics reported for various neat nanothermite and modified nanothermite compositions are given in Table 1. 

In conclusion, it is evident that the technology for the employment of nanothermite-based microthruster has evolved and matured rapidly. Various aspects of the development of nanothermite-based microthruster including synthesis of optimized nanothermite-based propellants, nozzle geometry of thruster engines, thruster array fabrication, and ignition parameters including ignition method, ignition energy and ignition delay have been thoroughly investigated. Among the various compositions investigated so far, 5%NC/GO/Al/KClO_4_ exhibits the best I_sp_ value of 203.2 s, which indeed is a significant achievement. 

## 8. Conclusions

Combustion properties such as burn rate, peak pressure and pressurization rate are not only characteristic of the nature of chemical composition but also geometry dependent. Reading the literature on combustion characteristics of NEMs such as measurements, it is evident that different geometries have been used. For example, open flame and partially confined and completely confined geometries have been employed in the case of burn rate measurements. Similarly, in the case of pressure–time measurements, different volumes of combustion chambers have been employed. Utilizing different test geometries makes it difficult to compare the combustion performance of different compositions reported in the literature. Therefore, it is pertinent to develop standards for the testing’ geometries and procedures. Standards may be defined as per the application’s need and desirability. Researchers must be encouraged to use these standards to enable direct comparison in the combustion performance between different compositions. Several research works reported in the literature have focused on reducing the ESD sensitivity of nanothermite-based NEMs. Very few studies have reported on reducing the impact and friction sensitivity. There is more scope for research on this front. 

Experimentally measured values of heat of reaction of various nanothermite systems are always lower than that of the values predicted by theory. Experimental observations by several research groups present enough evidence for the occurrence of a reaction between fuel and oxidizer in the condensed phase. Thus, the combustion reactions are diffusion-limited as the native Al_2_O_3_ shell passivates the metallic Al core, inhibiting the reaction rate severely. The native oxide shell also is a dead mass as far as the overall energy release is concerned and contributes nothing. There have been several approaches to passivate the surface of Al with energetic organic material such as perfluoroalkyl carboxylic acids, stearic acid, oleic acid, or energetic polymers such as PTFE, GAP, etc., or metals such as B, Ni, etc. Though combustion performance enhancement has been seen with some of these methods, the scalability of the process is an issue that needs to be addressed. An alternative attractive and viable proposition to passivation is the use of functionalized graphene with halogens such as iodine and fluorine. 

Processing of nanothermite-based energetic formulations in water is desirable for facilitating environmentally sustainable solutions. However, the challenge of inhibiting the reaction of Al nanoparticles with water still persists. Overcoming such a challenge will enable seamless integration with the processing methodology of secondary explosives that would lead to the facile synthesis of hybrid energetic formulations. Coating of Al nanoparticles with GAP is reported to be promising. There is scope for further research in this area. 

Among the several nanothermite-based EMs, graphene-based energetic materials show enormous promise to be a suitable candidate for various applications. Quantification of functional groups in FGS and correlation with synthesis methods is necessary to produce these NEMS with reproducible, and reliable combustion characteristics. Furthermore, quantitative correlation of the ignition parameters such as ignition energy, ignition delay and the method of ignition to the sensitivity parameters is essential. At the same time, the simplicity of the synthesis process, specifically in terms of processing duration, availability of ingredients, throughput, cost, scalability, and safety aspects at all times (during mixing of nanoscale ingredients, processing, storage and handling post-synthesis), environmental stability against moisture and heat and reduced sensitivity to external stimuli such as ESD, friction and impact, and cost are the key factors that will decide their utility in large scale applications. The mechanical integrity of nanoenergetic materials in various forms is another area of research that can pave the way for application development. Investigation of mechanical properties of nanothermite-based NEMs is another area of research opportunity. Exciting prospects exist for the computational design of novel energetic formulations via the utilization of a combination of machine learning and artificial intelligence methods using the available experimental data for training the algorithms accurately.

One of the important questions confronted often by the scientific community is the combustion performance of NEMs as a function of storage time. There have been very few studies reported in the literature. Recently, Wang et al. reported a detailed study on the storage stability of CuO/Al coated with 2.5 wt.% nitrocellulose prepared using the electrospray method [141]. The prepared NEMs were stored in a small glass vial, which was then placed in a glass desiccator kept at room temperature. It is therefore evident that the NEMs were not subjected to any harsh conditions such as hot or cool temperatures or humidity. The ignition and combustion performance were investigated as a function of storage time over a period of 13 months and correlated with the experimentally determined composition and morphology. It is satisfying to note that these composites exhibit stable ignition and combustion performance to a reasonable extent when stored in a controlled environment. However, it may be essential to conduct a systematic study on the ignition and combustion performance after subjecting the NEMs to different ambient conditions over a prolonged period of storage time. Since graphene-based NEMs are showing excellent potential with enhanced and tunable combustion performance, especially at reduced sensitivity to ESD, it is pertinent to study the aging behavior of these energetic formulations under harsh conditions. 

Similar to other materials that have been around in our lives for decades, the research of EMs is developing towards a more thorough understanding of scientific questions, especially the reaction mechanism, and a broader and more customized application in various environments. Studying the material from nanoscale structures, such as self-assembly and carefully designed structures, combined with state-of-the-art characteristic techniques will help us to promote both of them. On the side of fundamental understanding, in situ electron microscopy was used to figure out the morphology change in Al particles upon heating in different conditions [142,143], and high-speed micro pyrometry connected the nanoscopic reactions with microscopic (mm level) reaction observations [144,145] to elucidate macroscale combustion facts. On the side of applications, specific structures are designed to target practical needs in different conditions, such as in space [146], in water [147], and in areas unreachable using conventional methods [148,149]. Refreshing the well-known EMs with up-to-date materials, including versatile carbon nanomaterials, will ensure a promising scope of EMs in more applications.

## Figures and Tables

**Table 1:** Thrust characteristics of nanothermite mixtures.

Composition	ϕ Value	% TMD	I_sp_ (s)	I_sv_ (mN s/mm^3^)	Burn Duration (ms)	Reference
Al/CuO	1.6	56	20–25	Not reported	3	[135]
Al/CuO	1.0	55	20.2	0.5	4.5 ± 0.5	[136]
Al/Bi2O3	1.0	55	41.4	1.7	4.5 ± 0.5	[136]
2.5 wt.%NC/Al/Bi_2_O_3_	1.6	60	63.2	2.3	5–8	[138]
5 wt.% GO/Al/Bi_2_O_3_	1.0	55	71.5	Not reported	5	[81]
Al/KClO_4_	1.0	55	77.89 ± 1.61	0.48 ± 0.03	0.24 ± 0.02	[139]
5 wt.% GO/Al/KClO_4_	1.0	55	129.76 ± 2.13	0.75 ± 0.06	0.42 ± 0.03	[139]
5 wt.% GO/Al/KClO_4_	0.8	50	110.2 ± 2.3	2.8 ± 0.1	0.3 ± 0.1	[91]
5 wt.% GO/Al/KClO_4_	0.8	50	186–203	9.6 ± 0.1	2.4 ± 0.2	[91]

## Data Availability

All of the simulated data and input parameters used in the computational simulation work have been made available within the manuscript.
